# Diseases of the Maxillary Sinus

**Published:** 1864-07

**Authors:** William C. Horne


THE
### DENTAL REGISTER OF THE WEST.

VOL. XVIII.] JULY, 1864. [No. 7.

### Original Essays and Communications.

### DISEASES OF THE MAXILLARY SINUS.

An Essay read before the Brooklyn Dental Association, March 2d, 1864.

BY DR. WILLIAM C. HORNE.

The functions of the mouth and the important and intimate relations which it holds with other parts of the human body, are matters of deep interest to the dental profession; for a proper understanding of which, and skillful adaptation of means in restoring its diseased parts to a healthy condition, careful study and close observation are indispensable. The mouth is connected by its mucous membrane with the digestive and respiratory organs, and an unhealthy condition of the former will quickly exert a deleterious influence upon the latter. The nervous system is also brought into direct and often painful relations with it by the superior and inferior dental nerves.

Numerous diseases developed in the oral cavity come within the field of dental surgery, though most dentists confine themselves solely to the treatment of carious teeth. The great perfection attained by some in this branch of dental art, and therefore incumbent on all who would adorn their profession, renders the insertion of incomplete and defective plugs inexcusable in any one; and though great advances have been made, the most aspiring will still find room for progress. While we endeavor with clean hands to do clean work, we can with a very good grace impress upon our patients the necessity of keeping clean mouths.

Diseases of the dental pulp and periosteum have received much of our attention of late, and many interesting and instructive facts have been brought forward. The judgment of most of the members of this body probably is, that exposed pulps can not be treated better than by devitalization (by means of arsenic and creasote); to be followed by the removal of the dead nerve and filling of the tooth and fangs; while there still remain those who prefer to cap the exposed pulp (provided it be not inflamed), perhaps with a non-conducting substance, such as Hill's Stopping, filling the rest of the cavity securely, and trust to Providence for the result.

Salivary Calculus has been touched upon, with suggestions, from a distinguished source, for the restoration of the alveolar processes and renewed growth of the gums. Here is a field ripe for the harvest.

Alveolar Abscess has come in for a full share of attention; the subject exciting very great theoretical and practical interest. The incentive to the discovery and use of new modes of treatment is the laudable desire to preserve or restore to health the greatest number of those teeth which may be put under our care. The treatment of Alveolar Abscess by the former school of dental surgeons was almost wholly preventive, and in producing distinct evidence of permanent cures, by new and scientific methods, the profession is advanced a most important step in its upward career.

The Cavity of the Antrum and its Diseases is the subject of discussion for this evening; and first in order as to its location. The internal or nasal surface of the superior maxillary bone presents a large irregular opening into a cavity known to anatomists as the maxillary sinus, or cavity of the antrum. This opening is nearly closed in the articulated skull by the ethmoid, palate, lachrymal and inferior turbinated bones. The cavity of the antrum is somewhat triangular, corresponding in shape with the form of the body of the maxillary bone. Upon its inner wall are numerous grooves, lodging branches of the superior maxillary nerves, and projecting into its floor several conical processes, corresponding with the roots of the first and second molar teeth. Its interior surface is covered by a mucous membrane, continuous with that of the nose, which is known as the pituitary membrane. This cavity is subject to some of the most formidable diseases that ever befall the human body, and yet there are few that are not more fully written upon than this. While the simplest diseases of this cavity, if left to themselves, will take on the most alarming symptoms, those which are considered most dangerous from their inception may be reduced and fully cured by early attention. But a very great difficulty arises just here in the impossibility of determining, in many cases, the nature of the malady until it has progressed very far; for these diseases are very insidious and often exist for weeks or months before they are discovered; while their symptoms are so nearly similar as to cause much uncertainty as to their precise character. Aside from the danger which they occasion, they are extremely loathsome, from the fetid odor of the secretions, which, being prevented from escaping through the natural opening, in the nose, must needs pass through the cheek, or into the mouth, causing much distress to the patient. The state of the general health and habit of body do much to determine the malignity of these diseases. Any vice of body which weakens the vital energies of the system is a predisposing cause of disease; such are the mercurial, scorfulous, scorbutic and venereal, each of which may influence the character of the diseased action in a manner peculiar to itself.

Most writers on diseases of the antrum agree in ascribing them to a morbid condition of the teeth, gums, and alveolar processes, occasionally to mechanical violence, and very rarely to the presence of foreign bodies. The roots of the superior molars and bicuspids sometimes penetrate the cavity, and in other cases are only separated from it by a thin plate of bone; so that it could hardly be possible for protracted disease of the teeth and processes to exist and the antrum escape unscathed. For instance, it sometimes happens that an abscess formed in the socket of a superior molar discharges itself into this cavity. When this happens it gives rise to inflammation of the lining membrane, causing its secretions to become more or less vitiated.

In a healthy condition, the pituitary membrane secretes a slightly glutinous, transparent and inodorous fluid, by which it is constantly lubricated; but when inflamed, its secretions become serous and aeria exhaling an odor more or less offensive. This affection would be of little consequence were it not liable to give rise to a purulent condition of the secretions and subsequent engorgement of the sinus. The nasal opening having been closed, the fluids of the cavity gradually fill it, and finding no egress, they distend the surrounding bony walls, until the parts which are thinnest give way, and the secretions make a passage for their escape; sometimes through the cheek, or beneath it just above the alveolar ridge, or through the palatine arch, or by the sides of the roots of one or more of the teeth. From openings of this sort matter is sometimes discharged for years, while the disease in the antrum does not seem to undergo any change.

The curative treatment of muco-purulent secretions and engorgement of the maxillary sinus requires the evacuation of the impure matter, the removal of all causes of irritation, and the restoration of the lining membrane. An opening having been made, generally by the extraction of one of the molar teeth and the penetration of the floor of the antrum through its socket, it should be kept open, until healthy action is restored, by means of a canula fastened to a neighboring tooth. To prevent the matter from discharging continually into the mouth, and any food from getting into the sinus, the free end of the canula should be closed, except at intervals for the discharge of the accumulated secretions.

There exists a diversity of opinions as to the point at which the opening into the antrum should be made; but the concurrent testimony of many eminent names favors the one above mentioned.

A purulent condition of the fluids of the antrum is often followed by ulceration of the lining membrane, and sometimes by caries of the surrounding bones. The discharges vary from nearly white to yellow or black, according to the progress of the disease. A fungous ulcer is capable of spreading like a polypus, but is softer, and therefore makes less impression on the surrounding parts. If the ulcer be the result of a cancerous growth, the pain will be sharp and the discharges fetid, and any of the soft parts with which they come in contact will also ulcerate. The bones soften, the teeth loosen, a slow fever sets in, and life painfully wastes away. The cancerous tumor must be thoroughly eradicated, leaving only the bony parts, which should be carefully cauterized; after this they will exfoliate, when a chance for cure will be afforded, if the cancer has not penetrated too far. If the ulcer is of a fungous nature, the use of escharotics and sometimes the actual cautery become necessary to be repeated, until the fungous growth is completely destroyed. Injections and fomentations are applied to the ulcerated parts, and the general system kept up by tonics and such constitutional treatment as the individual case may require. These cases are regarded as almost incurable even when all the resources of surgery are put in requisition.

Tumors of the lining membrane and periosteal tissue are among the most fearful diseases of the antrum. They sometimes originate in the nose, frontal sinus, or ethmoidal cells, as well as in the antrum, and having filled the cavity, gradually distend its walls (which are softened under the pressure); the orbit is invaded, and the eye forced from its socket; the teeth drop out; the mouth is filled to its utmost, and the tumor protrudes in large masses from it. These tumors vary in appearance, structure and malignity; some presenting a smooth sensitive surface, of a healthy color; others being rough, painful and ulcerated, and varying from yellow to purple. Some are attached by a broad base, others by a mere peduncle. Their growth in the early stages is not very rapid, but it is accelerated as the adjacent parts become involved in the diseased action; the pain then becomes unbearable, and the patient finds relief only by the use of the most powerful anodynes. It is only in the earlier stages of these tumors that success may be expected from surgical treatment; for if not completely removed, reproduction will soon take place. The operation itself is one of the most formidable nature, calling for the use both of the knife and the actual cautery. While many perish by this lingering death, there are several successful cases recorded, where the sufferers were snatched from the very brink of the grave.

Having glanced at the diseases of the lining membrane and periosteum, let us now turn to those of the osseous walls of the antrum. The immediate cause of caries and necrosis of these parts is the destruction of their periosteum, by any of the diseases above named; while the softening of their walls is due to the action of the diseased secretions upon their calcareous particles. Complicated as they frequently are with other maladies, their cure is difficult and tedious; and when extended to such a degree as to involve the other bones of the face, a palliation of the symptoms is the most that can be safely predicted.

The walls of the antrum sometimes become the seat of a bony tumor called Exostosis. It consists of a deposition of ossific matter on the external surface of a bone, and the internal surface of its periosteum, to both of which it firmly adheres. This deposit varies from a spongy and cellular to a very dense structure. Exostoses sometimes attain an enormous size; one, recorded by a French surgeon, measuring at its greatest circumference twelve inches; this variety being of a spongy nature. Those of a denser structure are slower in growth. It is not uncommon for such, after having reached a certain size, to cease growing, and remain through life without giving rise to any very unpleasant consequences. The origin of this disease is probably dependent upon both local and constitutional causes; the presence of a venereal taint being considered specially favorable to its production. Constitutional treatment is generally recommended, with the use of mercurial medicines; but while its progress may be arrested, I find no evidence that an exostosis has ever been dispersed. It is not advisable, however, to attempt a removal unless the tumor is really painful and likely to become dangerous. When, however, the remedies proposed prove unsuccessful, the tumor should be removed, and the application of the actual cautery to any remaining portions will cause them to be exfoliated; after which if the exostosis is not complicated with any other disease, the restorative energies of nature will do what is required to complete the cure.

With this brief and general review of the ground to be gone over in this evening's debate, it remains for those present, of deeper knowledge and experience, to enlarge upon the subject—one of the deepest interest and highest importance—and to their eminent ability I commit it.

